# Mommy Meltdown: Understanding Racial Differences Between Black and White Women in Attitudes About Postpartum Depression and Treatment Modalities

**DOI:** 10.14740/jcgo664

**Published:** 2020-09-09

**Authors:** Talelia S. House, Eva Alnajjar, Madhuri Mulekar, Lisa B. Spiryda

**Affiliations:** a Department of Obstetrics and Gynecology, University of South Alabama College of Medicine, Mobile, AL 36688, USA; b Department of Mathematics and Statistics, University of South Alabama College of Medicine, Mobile, AL 36688, USA; c Department of Obstetrics and Gynecology, Phelps Hospital-Northwell, Zucker School of Medicine Hofstra/Northwell, Hempstead, NY, USA

**Keywords:** Pregnancy, Postpartum depression, Racial disparities, Social stigma of depression

## Abstract

**Background:**

Postpartum depression is a major public health problem, but limited information is available about risk factors and attitudes of minority women about postpartum depression. The objective of this study is to determine attitudes of minority women toward postpartum depression and treatment.

**Methods:**

In this prospective study at an academic resident and faculty clinic, 39 women (19 black and 20 white) at the 6-week postpartum visit completed a survey that was developed for this study to assess personal and family attitudes about postpartum depression in addition to the routinely distributed Edinburgh postnatal depression scale. The primary outcome variable was the presence of postpartum depression amongst minority women compared to other races. The secondary outcome looked at descriptors of attitudes about depression and treatment. Data were analyzed with Chi-square test for categorical data and Student’s *t*-test for continuous data.

**Results:**

Black and white participants were comparable in age, distribution of gestational age at birth, delivery type and pregnancy complications. The diagnosis of postpartum depression was not different in either population (two black and three white women; P = 0.667). Black participants were referred less frequently to counseling as treatment (5% vs. 30%; P = 0.052) but both black and white study participants viewed counseling as helpful (84% vs. 80%; P = 0.345). Black participants had a lower frequency of family history of depression (11% vs. 40%; P = 0.052) but both study groups were comfortable discussing the topic with their families, felt that their families were not ashamed of any social stigma about depression, and would be supportive of either counseling or medications as a treatment modality.

**Conclusions:**

Postpartum depression was common among our patients regardless of race. Most black and white women were willing to discuss depression with their families and accept treatment. Despite previous evidence to the contrary, black women stated that they were open to counseling as treatment for depression.

## Introduction

Depression is the most common mental health disorder affecting 17% of women of reproductive age, including 14% of women during pregnancy or postpartum [1-4]. The American College of Obstetricians and Gynecologists (ACOG) recommends that patients should be screened with a standardized, validated tool at least once during the perinatal period for depression and anxiety symptoms [2, 3]. Minority women are among the most affected by maternal depression in part due to contributors of socioeconomic disadvantage [4-7]. Maternal depression is associated with preterm birth, fetal growth restriction, low birth weight, and other postnatal complications [2]. Risks factors associated with the diagnosis of perinatal depression include a past history of depression, history of physical or sexual abuse including intimate partner violence, lack of financial or social support, and complications during pregnancy.

There have been many published studies examining preventive and early detection initiatives to assess the effects of screening, counseling, and pharmacotherapy on the prevalence of perinatal and postpartum depression. Counseling interventions were associated with a 39% reduction in the likelihood of perinatal depression when the incidence, prevalence, and scoring above the cutoff on a symptom severity scale were combined [8]. The Sertraline trial found that at 20 weeks postpartum, women taking sertraline had decreased depression recurrence and time to recurrence compared with those taking placebo [9]. Despite extensive global research about maternal depression, a recent comprehensive literature review [10] showed only eight studies that evaluated risk factors associated with maternal depression in black women [10-16]. One study reflected that black postpartum mothers are less likely than their white counterparts to accept prescription medication and mental health counseling, but are more likely to accept spiritual counseling [17].

Mommy Meltdown was birthed to address the important topic of depression in minority women, especially in the perinatal and postpartum periods. This study serves to highlight the sensitivity of this issue while unveiling the impact of the epidemic of untreated and unrecognized postpartum depression on minority women and their families. This study sheds light on the need for aggressive screening and access to care for minority women in efforts to address mental health issues.

We hypothesized that perinatal depression would be highest amongst black patients, and they would be open to treatment and view it positively. The goal of this study was to determine the prevalence of postpartum depression in our clinic population, compare prevalence as well as attitudes towards postpartum depression and treatment modalities between black and white women.

## Materials and Methods

### Study participants

This prospective study was approved by the University of South Alabama Institutional Review Board. It was conducted in compliance with ethical standards consistent with University of South Alabama policies on human subjects, as well as with the Helsinki Declaration. The study was conducted at University of South Alabama Obstetrics and Gynecology resident and faculty clinics. A total of 41 women completed the survey from July 2018 through March 2019 at their 6-week postpartum visit; two surveys were excluded from analysis because they failed to identify as black or white; a sample size of 2 in the “other” category was an insufficient sample size for statistical analysis. It is routine for all women to complete the Edinburgh postnatal depression scale (EPDS) regardless of study participation.

### Measures

At the 6-week postpartum clinic visit, participants were asked to complete the EPDS (10 items, with higher scores representing higher levels of depressive symptoms; total score: minimum, 0; maximum, 30). Any scores of 9 and above were flagged for the provider for further exploration of symptoms and suicidal ideation [18]. Study participants also completed the survey that was developed for this study to assess personal and family attitudes about postpartum depression (Supplementary Material 1, www.jcgo.org). This survey was developed personally by the authors and included three components: demographic information and pregnancy outcomes, family and personal history of depression, and depression treatment. The questions were all opinion-based and allowed patients to elaborate on each question (Supplementary Material 1, www.jcgo.org). The primary outcome variable was the presence of postpartum depression amongst minority women compared to others. The secondary outcome looked at descriptors of attitudes about depression and treatment.

### Statistical analysis

Data were analyzed with statistical software (JMP, SAS Institute, Cary, NC). Comparisons were evaluated with Chi-square test for categorical data and Student’s *t*-test for continuous data. Statistical significance was defined by P value < 0.05.

## Results

Average age of study participants was 25.8 years (range: 16 - 24) with 84.6% delivering greater than 36 weeks with a vaginal delivery rate of 84.5% and complication rate of 38.5% (Table 1). When the data were stratified by race, and there was no statistical difference between these basic clinical and demographic values (Table 1).

The mean EPDS was 9.29 for all study participants. The average score on the EPDS for black participants was 7.44 and 10.67 for white participants which was not statistically different (Table 2). The rate of postpartum depression was similar for both black and white study participants (11% and 15%; P = 0.667). Additionally, black and white study participants had equal rates of a history of depression and anxiety, and both groups equally indicated that their primary care physician and obstetrician discussed mental health issues with them. One interesting finding was that black women were less likely to be referred for counseling than white women (5% vs. 30%; P = 0.052). Contrary to what has been published in prior studies, both black and white women would consider counseling helpful (84% vs. 80%; P = 0.345) and viewed counseling as a positive experience (85% vs. 65%; P = 0.817). Both black and white study participants were treated with anti-depressive medications in the past and found that they were effective (67% vs. 71%; P = 0.880).

The next component of our survey was to examine how families viewed depression and treatment modalities (Table 3). Black study participants had lower rates of a family history of depression when compared to white study participants (11% vs. 40%; P = 0.052). There was no significant difference between rates of black families vs. white families in discussing depression (53% vs. 60%; P = 0.643) or viewing depression as a social stigma (5% in both groups). Black study participants did think that their family may view them differently with a diagnosis of depression (20% vs. 0%; P = 0.077; Table 3). We also examined if families would be supportive of a diagnosis of depression and what types of treatment modalities would be accepted by their families. Both groups felt that their families would be supportive of a depression diagnosis and would be supportive of the treatment for depression (> 90% in both groups; Table 3). Lastly, the majority of study participants regardless of race felt that families would likely accept both counseling and medications for treatment modalities (85.3%; Table 3).

## Discussion

Postpartum depression was present in our patient population regardless of race. Both black and white women were comfortable discussing depression with their providers (primary care provider (PCP), obstetrician) and perceived that their families as receptive to them receiving treatment for depression.

One of the more interesting findings in our study was that although black women were treated at a lower rate with counseling than white women (5% vs. 30%), black women were open to the idea of counseling as a form of treatment and replied at a slightly higher rate that it would be a positive experience in their lives (84% vs. 65%). This is in contrast to previously published studies. In addition to other studies, family support was perceived to be high and other social stigma was found to be infrequent in both black and white participants.

Based on these findings, black women in our clinic are receptive to counseling as a form of treatment for depression despite previous impressions from other studies, and should be encouraged by health providers to seek counseling and treatment for their diagnosis of depression. One study showed that overall postpartum depression therapy acceptance was high though black mothers were less likely than white mothers to accept prescription medication and mental health counseling, and more likely to accept spiritual counseling for postpartum depression treatment [17]. African American women have also reported that they relied on prayer and faith to help them through their mental pain [19]. Religion-based counseling appears to be valuable and may be an aspect of counseling that needs to be explored as an option for women. Although our study did not specifically discuss the different types of counseling, black women did appear to be receptive to traditional counseling modalities.

Previous studies have also shown that women may be reluctant to take pharmacological antidepressants during pregnancy and/or the postpartum as only 18% of depressed mothers seek treatment [20]. Only 26% of our patient population reported using antidepressant medications previously but women in both groups that received medications had successful results and their families would be supportive of antidepressive medications as a treatment modality.

ACOG recommends that patients be screened with a standardized, validated tool at least once during the perinatal period for depression and anxiety symptoms [2, 21]. Mothers with postpartum depression show decreased rates of breast-feeding as well as an increased risk for failure to thrive, accidents due to parental inattentiveness, and non-accidental trauma as a result of direct lack of interest in caring for the infant. Postpartum depression not only has an effect on the mother-child relationship but can affect the entire family, introducing such issues as domestic abuse and child neglect [22]. Healthcare providers should ensure that mothers have contact within 3 weeks after delivery to evaluate the patient’s mental well-being and complete further comprehensive assessment [21].

### Research implications

Our study shows that patients, especially minority patients, are open to treatment for depressive symptoms. This information can be used to further study this topic and evaluate outcomes of minority women who receive pharmacologic therapy only, counseling only, or both therapies in the sehing of postpartum depression. Our clinics do not currently have mental health counselors on hand for patient care which limits our management. Majority of patients are referred to an outside facility for further mental health management, which leaves us unable to determine if patients follow up for further care. Adding a mental health counselor to our staff for patients to interact with in the perinatal and postpartum periods would improve access to care in regards to mental health issue and give our patient more immediate options when they are symptomatic.

A strength of this study is in the realistic perspectives of patients provided by the surveys in both black and white participants. Limitations of the present study include frequency of incomplete surveys along with small number of participants in the study.

In summary, postpartum depression is a serious and common health issue regardless of race. However, women who have postpartum depression typically are receptive to receiving treatment for this condition and most of them have an impression that their families are not ashamed of social stigma regardless of race.

## Supplementary Material

Depression-survey **Suppl 1.** The survey of personal and family attitudes about postpartum depression.

## Figures and Tables

**Table 1.** Patient Demographics

	Total	Black	White
Survey participants	39	19	20
Average age (range)	25.8 (16 - 40)	25.7 (16 - 40)	26.1 (17 - 34)
Gestational age at birth			
>36 weeks	33 (84.6%)	17 (89.5%)	16 (80%)
32 - 36 weeks	5 (12.8%)	2 (10.5%)	3 (15%)
24 - 32 weeks	1 (2.6%)	0	1 (5%)
Mode of delivery			
Vaginal	33 (84.6%)	16 (84.2%)	17 (85%)
Cesarean section	6 (15.4%)	3 (15.8%)	3 (15%)
Nursery type			
Newborn	34 (87.2%)	18 (94.7%)	16 (80%)
NICU	5 (12.8%)	1 (5.3%)	4 (20%)
Complications			
Preeclampsia	5	1	4
Diabetes mellitus	3	2	1
Fibroids	1	1	0
Pancreatic issues	1	1	0
Other undefined	1	1	0
Dystocia	1	0	1
PPROM	1	0	1
Postpartum cardiomyopathy	1	0	1
Postpartum hemorrhage	1	0	1

NICU: neonatal intensive care unit; PPROM: preterm premature rupture of the membranes.

**Table 2.** Patient Attitudes About Mental Health Issues and Treatment Modalities

	Black	White	P value
Average EPDS score	7.44	10.67	0.839
Diagnosed with postpartum depression, % (n)	11% (2)	15% (3)	0.667
Previous history of depression or anxiety, % (n)	37% (7)	40% (8)	0.839
Discussed mental health issues with PCP, % (n)	26% (5)	45% (9)	0.249
Discussed mental health issues with obstetrician, % (n)	68% (13)	70% (14)	0.522
Referred for counseling, % (n)	5% (1)	30% (6)	0.052
Counseling helpful, % (n)	84% (16)	80% (16)	0.345
Counseling positive experience, % (n)	84% (16)	65% (13)	0.817
Ever treated with medication, % (n)	26% (10)	35% (7)	0.105
Medications effective, % (n)	67% (2)	71% (5)	0.88

EPDS: Edinburgh postnatal depression scale; PCP: primary care provider.

**Table 3.** Family Attitudes About Depression and Treatment Modalities

	Black	White	P value
Family history of depression, % (n)	11% (2)	40% (8)	0.052
Family discussed depression, % (n)	53% (10)	60% (12)	0.643
Family ashamed of social stigma of depression, % (n)	5% (1)	5% (1)	0.916
Family would view you different with diagnosis of depression, % (n)	20% (3)	0% (0)	0.077
Family would be supportive of depression diagnosis, % (n)	94.7% (18)	90% (18)	0.579
Family supportive of treatment for depression, % (n)	100% (19)	90% (18)	0.157
Supportive of what types of treatment?			
Medications only, % (n)	11.1% (2)	6.3% (1)	
Counseling only, % (n)	5.6% (1)	6.3% (1)	
Both, % (n)	83.3% (15)	87.5% (14)	

## Data Availability

The data supporting the findings of this study are available from the corresponding author upon reasonable request.
