# Supplementary material for: Mommy Meltdown: Understanding Racial Differences Between Black and White Women in Attitudes About Postpartum Depression and Treatment Modalities
Source: J Clin Gynecol Obstet. Author manuscript; Available in PMC 2021 Mar 16. (PMC7962419; doi:10.14740/jcgo664)
Supplement: Depression-survey — Suppl 1. The survey of personal and family attitudes about postpartum depression. [file NIHMS1676028-supplement-Depression-survey.pdf]

EPDS Score: ___ / 30

USA HEALTH

MOMMY MELTDOWN: A LOOK AT PREVALENCE OF POSTPARTUM DEPRESSION AND ATTITUDES OF MINORITY WOMEN TOWARD DEPRESSION TREATMENT CONSIDERATIONS

1. **At what gestational age did you deliver your baby?**
   - **a.) <24 weeks**
   - **b.) 24-32 weeks**
   - **c.) 32-36 weeks**
   - **d.) >36 weeks**

2. **Did you deliver vaginally or by cesarean section?**
   - **a.) Vaginal (Normal)**
   - **b.) Cesarean Section**

3. **Were there any complications during your pregnancy/delivery?"**
   - **a.) Preeclampsia**
   - **b.) Diabetes**
   - **c.) Sickle Cell Anemia**
   - **d.) Growth Restriction (small-sized baby)**
   - **e.) Other: Please Specify below.**

4. **Which nursery was your baby taken to after delivery?**
   - **a.) Newborn (Normal)**
   - b.) **NICU (Neonatal Intensive Care Unit)**

General Questions

5. **Have you ever been diagnosed with depression or anxiety?**

6. **Have you ever been diagnosed with postpartum depression?**
   - **a.)** If **YES, "When were you diagnosed?"**
   - **b.)** NO

7. **Has anyone in your family ever been diagnosed with depression?"**
   If **YES,**
   a. Who in your family was diagnosed?

USA Institutional Review Board	
Approved:	8/1/2018
Expires:	
IRB number:	18-280/1235524-1

Survey No. ___

b. **When were they diagnosed?**

c. **Did they undergo counseling?**

d. **Did they take anti-depressive medications?**

8. **Do you see your PCP (Primary Care Doctor) regularly?**

If **YES,**

a. **How often do you see this doctor?**

b. **Does this doctor discuss/inquire about mood changes or mental health issues?**

If **NO**,

c. **Where do you primarily receive your regular healthcare?**

d. **Does this doctor question you about mood changes or mental health issues?**

9. **When you are pregnant, does your OB/GYN ask you about mood changes or mental health issues?"**
CIRCLE YES or NO

If **YES,**

a. **How often or when does this doctor ask throughout the pregnancy?**

b. **Do you wish he/she would ask more often?"**

Counseling

10. **Do you think counseling would be helpful for patients suffering with depression?**
CIRCLE YES or NO

11. **Do you think counseling would negatively or positively affect your life?**
CIRCLE YES or NO

USA Institutional Review Board
Approved: 8/1/2018
Expires:
IRB number: 18-280/1235524-1

12. Have you personally been treated for depression with counseling?
CIRCLE YES or NO

Anti-depression Medications

13. Have you ever been treated with anti-depressant medications?
CIRCLE YES or NO

a.) What medication did you take?

b.) How long did you take the medication?

c.) Do you feel that the medication helped you and/or resolved your symptoms?

d.) Have you taken any other medications to treat your depression (NOT anti-depressants)?

Family and Depression

14. Do you and your family discuss depression or mental health issues openly?
CIRCLE YES or NO

15. How does your family/community view depression?

16. Do you feel that your family is ashamed of the social stigma surrounding depression or mental illness?
If YES, please explain further why

USA Institutional Review Board
Approved: 8/1/2018
Expires:
IRB number: 18-280/1235524-1

17. **Do you feel that your family and friends will view you differently if you were diagnosed with depression?**

If **YES, please explain further why**

18. **Do you feel that your family will be supportive of you if you were diagnosed with depression?**

CIRCLE YES or NO

19. **Do you feel that your family will be supportive of treatment for depression?**

CIRCLE YES or NO

If **YES,**

a) **Medication therapy? YES or NO**

b) **Counseling? YES or NO**

c) **BOTH? YES or NO**

AGE:

RACE/ETHNICITY:

USA Institutional Review Board
Approved: 8/1/2018
Expires:
IRB number: 18-280/1235524-1

Survey No. ___
